# Macrophages but not Astrocytes Harbor HIV DNA in the Brains of HIV-1-Infected Aviremic Individuals on Suppressive Antiretroviral Therapy

**DOI:** 10.1007/s11481-018-9809-2

**Published:** 2018-09-07

**Authors:** Allen Ko, Guobin Kang, Julian B. Hattler, Hadiza I. Galadima, Junfeng Zhang, Qingsheng Li, Woong-Ki Kim

**Affiliations:** 1 Department of Microbiology and Molecular Cell Biology, Eastern Virginia Medical School, Norfolk, VA USA; 2 Nebraska Center for Virology, School of Biological Sciences, University of Nebraska-Lincoln, Lincoln, NE USA; 3 Graduate Program in Public Health, Eastern Virginia Medical School, Norfolk, VA USA; 4 Present Address: School of Community and Environmental Health, College of Health Sciences, Old Dominion University, Norfolk, VA USA; 5 Department of Human Anatomy, Xi’an Medical University, Shaanxi, China

**Keywords:** Antiretroviral therapy, Brain, HIV-1, HIV-associated neurocognitive disorders, Macrophage, Reservoir

## Abstract

The question of whether the human brain is an anatomical site of persistent HIV-1 infection during suppressive antiretroviral therapy (ART) is critical, but remains unanswered. The presence of virus in the brains of HIV patients whose viral load is effectively suppressed would demonstrate not only the potential for CNS to act as an anatomical HIV reservoir, but also the urgent need to understand the factors contributing to persistent HIV behind the blood-brain barrier. Here, we investigated for the first time the presence of cells harboring HIV DNA and RNA in the brains from subjects with undetectable plasma viral load and sustained viral suppression, as identified by the National NeuroAIDS Tissue Consortium. Using new, highly sensitive in situ hybridization techniques, RNAscope and DNAscope, in combination with immunohistochemistry, we were able to detect HIV-1 in the brains of all virally suppressed cases and found that brain macrophages and microglia, but not astrocytes, were the cells harboring HIV DNA in the brain. This study demonstrated that HIV reservoirs persist in brain macrophages/microglia during suppressive ART, which cure/treatment strategies will need to focus on targeting.

**Electronic supplementary material:**

The online version of this article (10.1007/s11481-018-9809-2) contains supplementary material, which is available to authorized users.

## Introduction

The combination antiretroviral therapy (ART) has converted a life-threatening human immunodeficiency virus (HIV) infection into a chronic disease. Nevertheless, life-long treatment is required for those with this debilitating “incurable” disease. While much effort has been focused on better understanding the nature of HIV cellular latent reservoir in peripheral blood cells and lymphoid tissues, non-lymphoid tissues including brain have not been rigorously investigated. Furthermore, despite the widespread use of ART, HIV-associated neurocognitive disorders (HAND) remain surprisingly common (Masliah et al. 2000; Sacktor et al. 2002; McArthur et al. 2003; Becker et al. 2011; Harezlak et al. 2011). Even in individuals on successful HAART with undetectable plasma viral load, HAND is still observed (Baeuerle et al. 2005; Simioni et al. 2010; Bingham et al. 2011; Cysique and Brew 2011). The basis of HAND in virally suppressed individuals is unclear, and one possibility is that a cryptic HIV replication in the brain may occur during suppressive antiretroviral therapy (ART); however, it remains to be tested whether the human brain is an anatomical site of persistent HIV-1 infection and/or ongoing low-level replication during suppressive ART. Moreover, the cell types in brain that persistently harbor HIV DNA and RNA in HIV patients on suppressive ART with undetectable viral load in blood and cerebrospinal fluid (CSF) are not established. Studies on HIV reservoirs in the brain have been largely hampered by the scarcity of autopsy brain tissue from HIV-infected individuals who had been on ART and achieved viral suppression for extended periods before their HIV-unrelated deaths. Furthermore, such studies have also been hampered by the lack of sensitive technologies for detecting the presence of low-copy viral RNA (vRNA) and DNA (vDNA) in specific cell types.

The National NeuroAIDS Tissue Consortium (NNTC) identified a limited number of cases on ART with well-documented, sustained control of HIV-1 (“Virally Suppressed Cases”). Exploiting this unique cohort, we used highly specific in situ hybridization (ISH) with single-copy sensitivity (RNAscope and DNAscope) to detect and quantify HIV-1 vRNA and vDNA in the brain and determined the cell types harboring vRNA and vDNA. We were able to detect persistent HIV-1 DNA in the brains of all 16 virally suppressed cases. In addition, we found that macrophages, including CD206^+^ perivascular macrophages (PVM) and some CD68+ microglia, harbored vDNA. Interestingly, no evidence for infection of astrocytes was indicated by absence of vDNA in astrocytes. Of note, we found, in some individuals (6/16) on suppressive ART with undetectable viral loads in blood and CSF, the presence of isolated focal vRNA in the brain. Together, these findings suggest that macrophages and microglia are the HIV cellular latent reservoir in brain, and that cure/treatment strategies are needed to focus on targeting the macrophage/microglial viral reservoir in the brain.

## Materials and Methods

### Study Subjects

The cases for this study were selected from over 900 human autopsied brain specimens in the NNTC. The following 4 groups and a total of 29 cases were examined (Table 1): Group 1 (*N* = 6) – HIV-1 uninfected subjects with minimal non-diagnostic abnormalities at autopsy (HIV-); Group 2 (*N* = 7) – HIV-1-infected subjects with encephalitis (HIVE); Group 3 (*N* = 8) – HIV-1-infected, virally suppressed subjects with HAND (HAND); Group 4 (N = 8) – HIV-1-infected, virally suppressed subjects with no functional impairment (No HAND). The further selection criteria for Groups 3 and 4 were as follows:

- Group 3: includes only HIVE-negative, suppressed cases with probable/possible mild neurocognitive disorder or HIV-associated dementia at last pre-mortem visit, but excludes hemorrhage dura/leptomeninges and bacterial parenchymal infection at autopsy.
- Group 4: includes only HIVE negative, suppressed cases with normal or asymptomatic neurocognitive impairment neurocognitive diagnosis at last pre-mortem visit, but exclude hemorrhage dura/leptomeninges and bacterial parenchymal infection at autopsy.

**Table 1:** Patient characteristics

ProjID	Category	PMI	Age at death	Sex	R	Risk	CD4	Plasma vl	CSF vl	Brain pathology	Global deficit score
01965	Uninfected	11	63	F	W	–	–	–	–	Abn-unspecified	–
02711	Uninfected	29.7	54	M	B	–	–	–	–	normal	–
02105	Uninfected	21.27	51	M	H	–	–	–	–	normal	–
01640	Uninfected	19	63	M	H	–	–	–	–	normal	–
01902	Uninfected	18.5	21	M	H	–	–	–	–	normal	–
01923	Uninfected	6.5	61	M	H	–	–	–	–	Abn-Focal Infarct	–
00039	HIVE	8	46	M	W	Hom-sx	15	167,143	11,908	HIVE	4.5
00284	HIVE	4	36	M	H	Hom-sx	16	195,269	–	HIVE	–
00488	HIVE	5.5	42	M	W	Hom-sx	99	6940	750,000	HIVE, MGNE	–
01555	HIVE	48	47	M	W	Hom-sx	9	210,000	–	HIVE	–
01580	HIVE	4	50	M	H	IVDU	1	58,419	–	HIVE	–
01598	HIVE	27	45	F	B	IVDU	6	–	–	HIVE	–
02390	HIVE	17	63	F	B	Het-sx	25	730,085	–	HIVE	–
01374	Virally Supressed with HAND	22.5	54	M	W	Hom-sx	491	85	50	minimal	1.79
02398	Virally Supressed with HANDI	4.5	64	M	B	Hom-sx	1043	40	na	Leukoencephalopathy	–
00630	Virally Supressed with HAND	18.5	61	M	B	Het-sx, IVDU	271	50	50	Abn-unspecified	0.57
00699	Virally Supressed with HAND	46.5	59	M	B	Hom-sx	491	50	–	Aseptic Leptomeningitis, Focal infarct,	0.71
02035	Virally Supressed with HAND	18.23	50	M	H	Hom-sx	300	20	–	Abn-Alzheimer/Focal Infarct	2.67
01986	Virally Supressed with HAND	8.5	69	M	W	Hom-sx	355	20	60	ischemic	2.62
00006	Virally Supressed with HAND	11.5	62	F	B	Het-sx	392	50	50	ischemic	0.15
00009	Virally Supressed with HAND	11	50	F	W	Het-sx, IVDU	791	50	50	Abn-unspecified	0.6
02104	Virally Suppressed no HAND	6	47	M	H	Hom-sx	61	48	–	Abn-Focal Infarct	0.36
01495	Virally Suppressed no HAND	7	39	M	B	Het-sx	112	40	–	ischemic	0.21
00697	Virally Suppressed no HAND	21	66	M	W	Hom-sx	798	50	–	Abn-Focal Infarct	1.21
00522	Virally Suppressed no HAND	24	66	M	W	Hom-sx	465	50	50	normal	0.07
00658	Virally Suppressed no HAND	6	52	M	W	Hom-sx	78	50	50	normal	0
00719	Virally Suppressed no HAND	20	44	M	B	IVDU	219	50	50	ischemic	0.86
01878	Virally Suppressed no HAND	8.5	63	M	W	Hom-sx	133	50	50	ischemic	1.79
02087	Virally Suppressed no HAND	14.5	51	M	W	Hom-sx	223	50	139	Abn - ChronicHTN	0.77

There were only 11 cases available for group 3 and 12 available for group 4. The NNTC sites randomly assigned 8 to each group. Groups 1 and 2 were matched as best as possible with respect to age, sex, and race; there was no statistically significant difference in demographic parameters (Supplementary Table 1). Postmortem intervals were all under 48 h. Please visit the NNTC website (https://nntc.org/cohorts/virally-suppressed-cases) for more information. Users can search for a case by using a PRJID in Table 1.

### Neuropsychological Assessment

All virally suppressed subjects received a 2-to-3-h battery of neuropsychological (NP) tests. The tests are fully described elsewhere (Fischer-Smith et al. 2001; Woods et al. 2004), and also listed on the NNTC website. The NP battery included tests of premorbid functioning, speed of information processing, attention/working memory, verbal fluency, learning and memory, abstraction/executive functioning, motor skills, and assessments of everyday functioning and mood. Raw test scores were converted to age and demographically corrected T scores, which were then used to compute a deficit score that ranged from 0 (T score > 39, no impairments) to 5 (T score < 20, severe impairment). Average deficit scores are computed to yield an overall level of NP performance [global deficit score (GDS)] (Blackstone et al. 2012). A diagnosis of HAND at the final pre-mortem visits was made by American Academy of Neurology criteria that were modified by the Frascati recommendations (Janssen et al. 1991; Antinori et al. 2007).

### HIV vDNA and vRNA Detection Using DNAscope and RNAscope ISH

HIV DNA and RNA in brain tissues were detected in situ using sense and antisense probes from the RNAscope® ISH probe-V-HIV1-clade B (Cat# 416111, Advanced Cell Diagnostics) and RNAscope® 2.5 HD assay-Red (Advanced Cell Diagnostics) for color development, respectively. The RNAscope® negative control probe-DapB (Cat# 310043, Advanced Cell Diagnostics) was used as negative control. The DNAscope and RNAscope ISH was conducted according to our previously reported protocol (Yuan et al. 2017). The tissue sections, after RNAscope ISH or DNAscope ISH was performed, were digitized with Aperio CS2 Scanscope. For quantitative image analysis of vRNA- or vDNA-positive cells, a positive pixel count algorithm in Aperio’s Spectrum Plus analysis program (version 9.1; Aperio ePathology Solutions) was used as we previously reported (Wang et al. 2014).

### Determining the Cell Types of vRNA^+^ and vDNA^+^ Cells Using RNAscope/DNAscope ISH in Combination with Immunohistochemistry

The tissue sections of vRNA- or vDNA-positive tissue sections post ISH were digitized with Aperio CS2 Scanscope, and then the coverslips of these tissue sections were removed by soaking tissue slides in xylene overnight. The tissue sections were rehydrated for immunohistochemistry (IHC) conducted as we reported (Li et al. 2009). Briefly, mouse-anti-human CD68 monoclonal antibody (KP1, 1:200, Leica), mouse-anti-human CD206 monoclonal antibody (5C11, 1:500, Abnova), or mouse-anti-GFAP monoclonal antibody (ASTRO6, 1:100, Thermo Scientific) was used as the primary antibody. Mouse IgG isotype control antibody was used as negative control. EnVision anti-mouse HRP polymer kit (Cat#: K400111–2, Agilent Dako) and substrate diaminobenzidine (DAB) were used to visualize antibody staining signals as brown. After counterstained with hematoxylin, tissue sections were digitized with Aperio CS2 Scanscope and were analyzed using Aperio’s Spectrum Plus analysis program (version 9.1; Aperio ePathology Solutions) to separate into single channels of vRNA, vDNA, or antibody staining signals. The tissue sections on slides after combined DNAscope ISH with IHC were also reviewed with Olympus Cell-Sens software (1.18) after images were captured with DP72 CCD camera at 60×/1.25 Oil Iris using Olympus microscope. To further determine whether astrocytes support HIV productive infection, fluorescent RNAscope ISH was combined with immunofluorescent staining of GFAP. Donkey anti-mouse IgG conjugated with Alexa Fluor 647 (Cat#: A-31571, Thermo Fisher Scientific) was used as a secondary antibody and cell nuclei were counterstained with DAPI. A Nikon A1R-TiE live cell imaging confocal system was used to visualize and capture images of stained samples.

### SAMHD1 IHC

IHC for SAMHD1 was done on human brain tissues as we previously reported (Lindgren et al. 2018)

### Statistical Analysis

Descriptive statistics of the cohort data were presented as mean (standard deviation) for continuous variables and frequency (percent) for the categorical variables (Supplementary Table 1). Comparisons of the HIV-infected groups (G2, G3, and G4) within and across each of the three brain regions with respect to HIV vDNA were assessed using a two-way ANOVA. Interaction effect between HIV-infected groups and the brain areas was systematically considered and retained based on its significance. Tukey pairwise multiple comparison was used for the post hoc-analysis. Finally, regression and Pearson correlation analyses were used to examine the relationship between HIV vDNA+ cells with GDS and SAMHD1+ cells in specific brain regions. All statistical analyses were performed using SAS version 9.4 (SAS Institute, Inc., Cary, NC) and assessed with a statistical significance level of alpha = 0.05.

## Results

We set out to determine whether brain harbors HIV vDNA or vRNA or both in the HIV-1-infected aviremic individuals who were on suppressive ART, as well as what type of cells harbor vDNA in those subjects. The NNTC identified 44 (now 56) cases of HIV-infected aviremic subjects who were on suppressive ART and donated the tissues including the brain. From this rare resource we obtained 16 such cases with available cognitive characterization to further stratify by HAND status (8 with HAND and 8 with no HAND). In addition, we included HIV-infected viremic individuals with encephalitis (HIVE group; *n* = 7) and HIV-1 uninfected individuals as controls (Table 1).

We studied 3 brain regions, frontal white matter (FWM), basal ganglia (BG) and corpus callosum (CC), of individuals in all four above-described groups. We used RNAscope and DNAscope ISH to detect vRNA and vDNA, respectively.

**Fig. 1:**
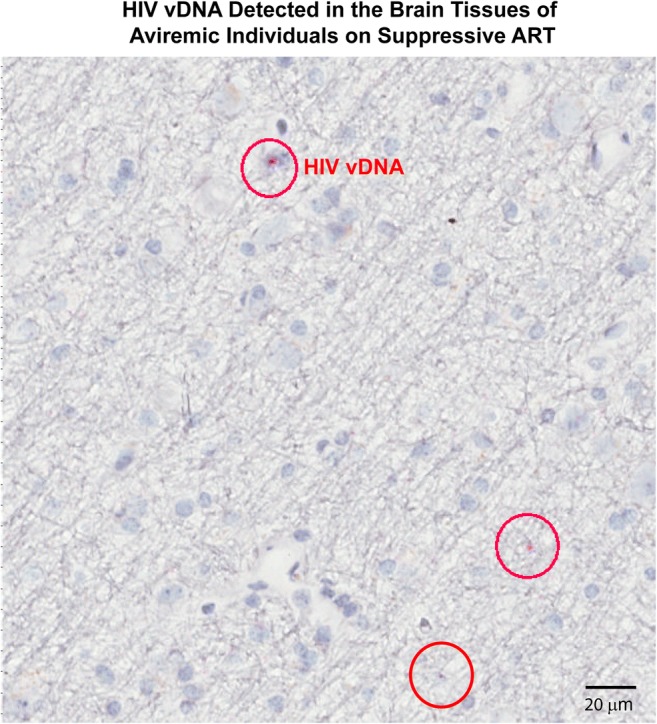
Representative image of HIV vDNA+ cells detected in the brain tissues of HIV infected aviremic individuals on suppressive ART. HIV vDNA-positive cells (a discreet red dot in a nucleus, red circle; virally suppressed subject 00009, FWM) were detected using sense probes from the RNAscope® ISH probe-V-HIV1-clade B and RNAscope® 2.5 HD assay-Red as a substrate (Advanced Cell Diagnostics). Cell nuclei were counterstained with hematoxylin

**Fig. 2:**
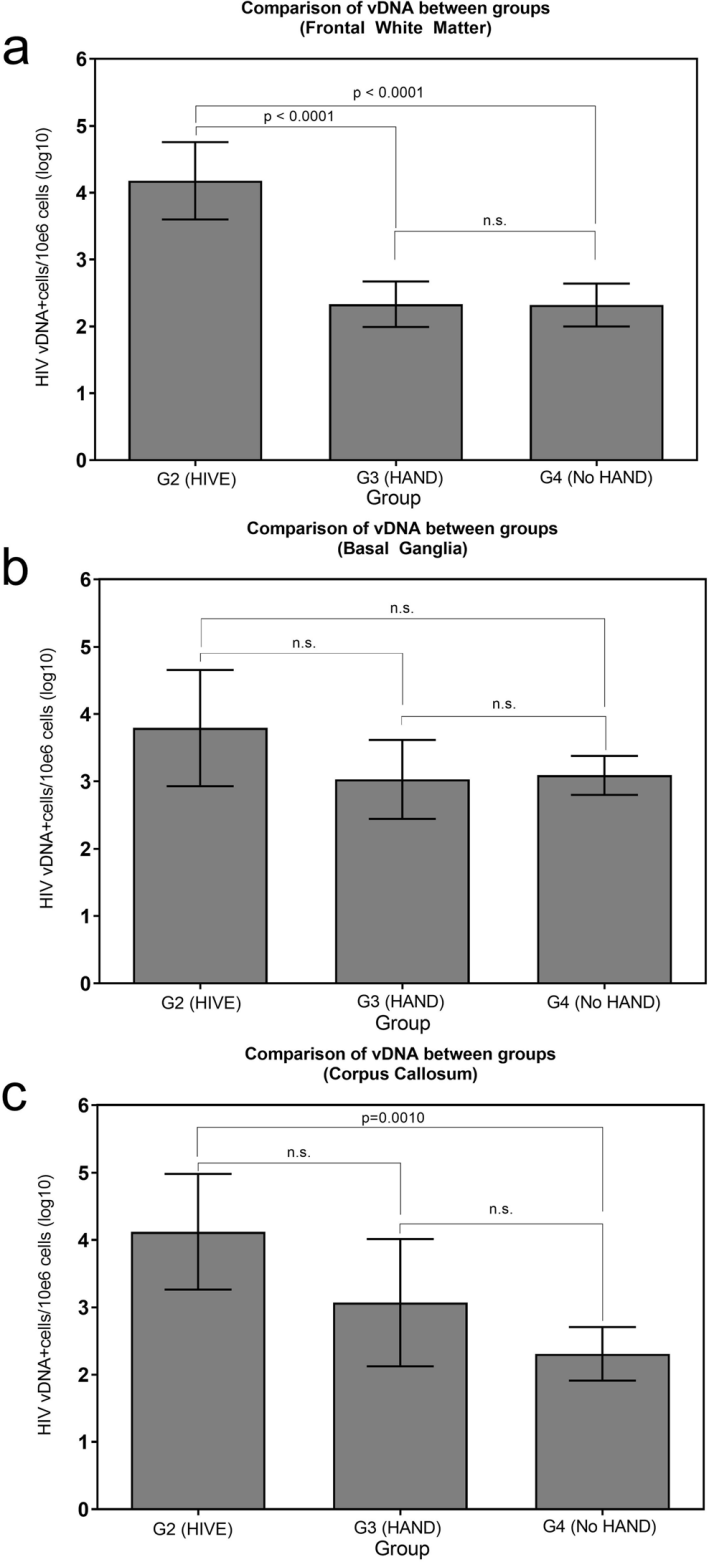
Comparison of vDNA levels between HIV-infected groups by brain area. We compared the distribution of viral DNA (vDNA) in three groups (G2 HIVE, G3 HAND, and G4 No HAND) through a two-way ANOVA with Tukey multiple comparison test. **a** shows frontal white matter (FWM), **b** basal ganglia (BG), and **c** corpus callosum (CC) DNA. HIVE: human immunodeficiency virus encephalitis; HAND; HIV-associated neurocognitive disorders

We first examined HIV-1 DNA in all three brain regions using DNAscope ISH. HIV vDNA signals (red puncta) were consistently detected in all individuals from the three HIV-infected groups including those who were virally suppressed (Fig. 1). We then quantified the frequency of vDNA+ nuclei in the entire section normalized with the total number of hematoxylin-counterstained nuclei. HIV vDNA^+^ cells were found in all three brain regions. A two-way ANOVA with interaction effect between brain areas and groups was used to compare HIV vDNA+ cell numbers. While the interaction effect between brain areas and groups was significant (*p* = 0.0248), we performed a post-hoc analysis using Tukey test. The means of HIV vDNA+ cell numbers were compared between groups within each brain area as depicted in Fig. 2. Virally suppressed subjects with or without HAND had significantly fewer HIV vDNA+ cells in the FWM compared to the subjects with HIVE (Fig. 2a; *p* < 0.0001). However, no statistically significant difference in the number of HIV vDNA+ cells in the BG was found across the groups (Fig. 2b, all *p*-values >0.3). Likewise, in CC, virally suppressed subjects with HAND had no significant difference in the number of HIV vDNA+ cells compared to the subjects with HIVE, although virally suppressed subjects with no HAND had significantly fewer HIV vDNA+ cells than those with HIVE (Fig. 2c; *p* < 0.001).

**Fig. 3:**
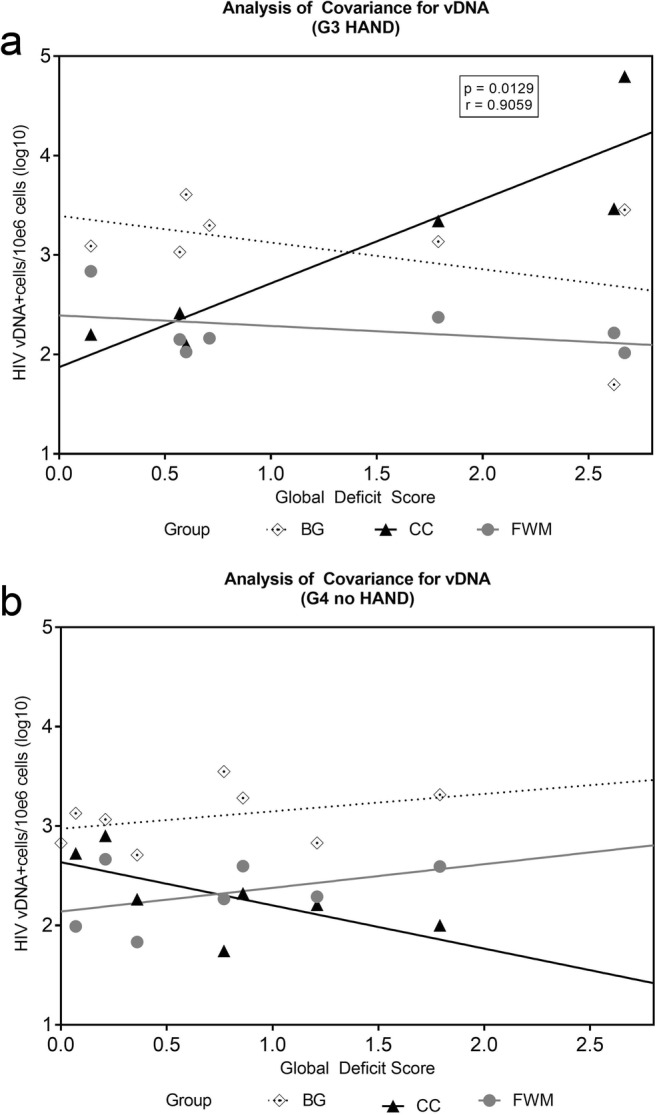
Correlation of Global Deficit Score (GDS) to HIV vDNA in virally suppressed groups G3 and G4. **a** G3 (HAND) group, and **b** G4 (No HAND) group. The slopes were not statistically significant except for one slope with CC in G3 (a; *r* = 0.9059, *p* = 0.0129)

While there was no difference in the number of vDNA+ cells between virally suppressed patients with HAND and those without HAND, we sought to investigate the relationship between HIV DNA levels and global deficit score (GDS). When we examined the relationship between the number of vDNA+ cells in specific brain regions and GDS among virally suppressed subjects, we found that there was a strong positive correlation between the number of HIV vDNA+ cells in the CC and GDS among virally suppressed subjects with HAND (Fig. 3; *r* = 0.906, *p* = 0.0129). This suggests that the presence of HIV-1 DNA in the brain, specific to particular regions including corpus callosum, may be important in inducing neurocognitive impairment.

**Fig. 4:**
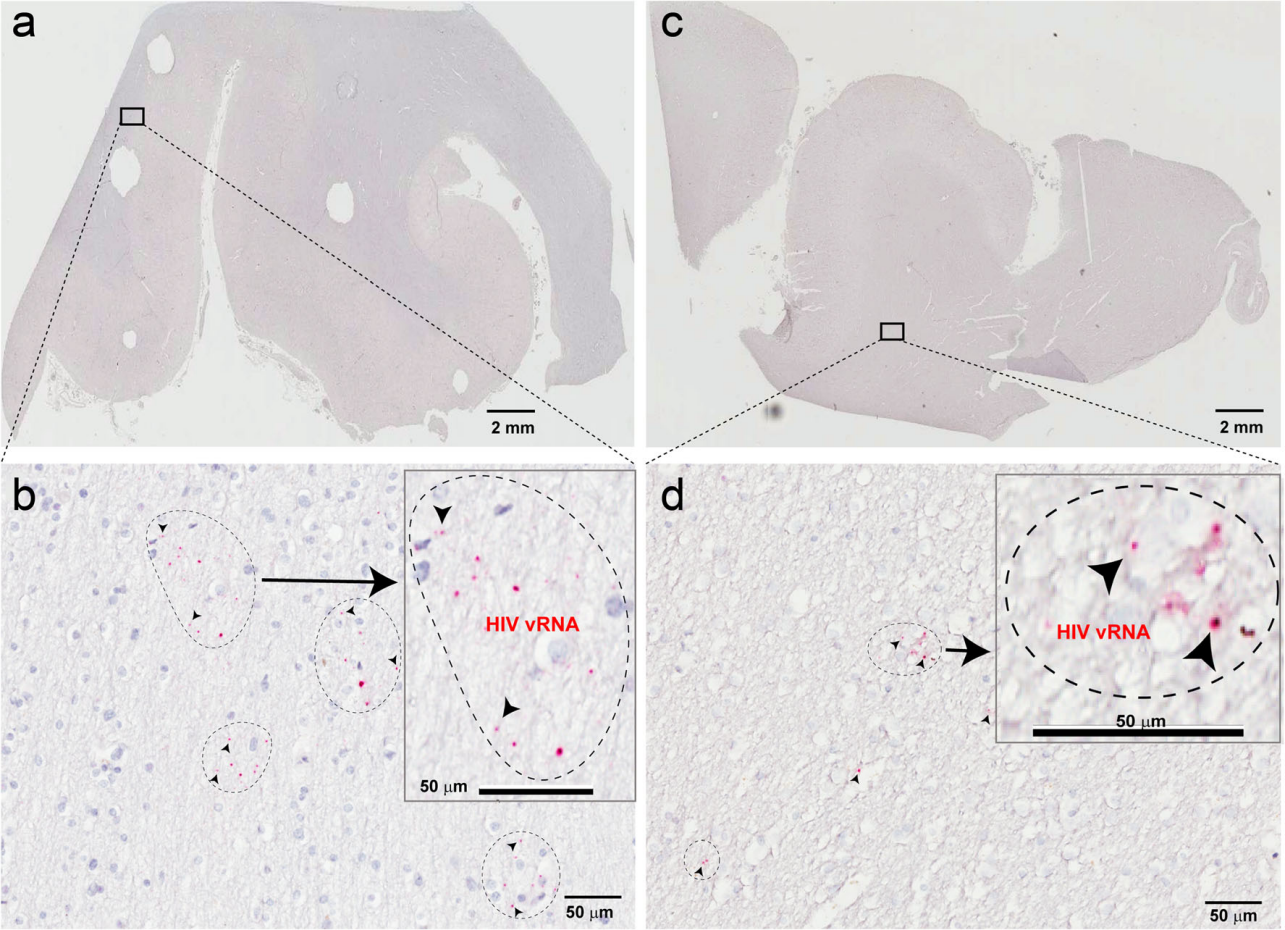
Small clusters of isolated vRNA signals were detected in the brain tissues of six HIV-1 suppressed aviremic individuals from HAND and No HAND groups using RNAscope ISH. HIV-1 RNA signals morphologically appear as cell-free virions (discrete red puncta, arrows). **a** low magnification of FWM and CC tissues from individual 00009 from HAND group, and **b** magnified image from the inset in the panel a, in which cell-free virion derived signals (discrete red dots) were visible; **c** low magnification of FWM and CC tissues from individual 01878 from the No HAND group, and **d** magnified image from the inset in the panel c, in which cell-free virion derived signals (discrete red dots) were visible

We next examined HIV vRNA in all three brain regions of all individuals from the three HIV-infected groups, including those who were virally suppressed. Abundant HIV RNA was readily detected in all the brain regions from HIVE group (Supplementary Fig. 1). We can distinguish cell-free vRNA (virion, Fig. S1b, black arrowheads) from cell-associated vRNA using the single-copy sensitive RNAscope ISH. As shown in Fig. S1, vRNA in the brains of HIVE group exists mainly in cell-associated form around small blood vessels. We did not detect any in situ signals in the subjects from negative control group using HIV-1 sense or antisense probes, and we also did not detect any in situ signals from the subjects of HIVE group using negative control probe (data not shown). In the majority of the virally suppressed aviremic subjects, we did not detect vRNA signals. However, we detected small clusters of isolated vRNA signals, which were infrequent and very focal, in 3 of 8 individuals from HAND group (01986, 00006, 00009) and 3 of 8 individuals from No HAND group (00658, 01878, 02087) (Fig. 4). Of note, vRNA signals morphologically appear as discrete red puncta representing cell-free virions (Fig. 4b & d, arrowheads), which differ from vRNA signals in HIVE group where abundant HIV vRNA signals were readily apparent in all the brain regions (Fig. S1) and the entire cytoplasm of the infected cells was filled with strong red puncta making individual puncta impossible to discriminate (Fig. S1b).

**Fig. 5:**
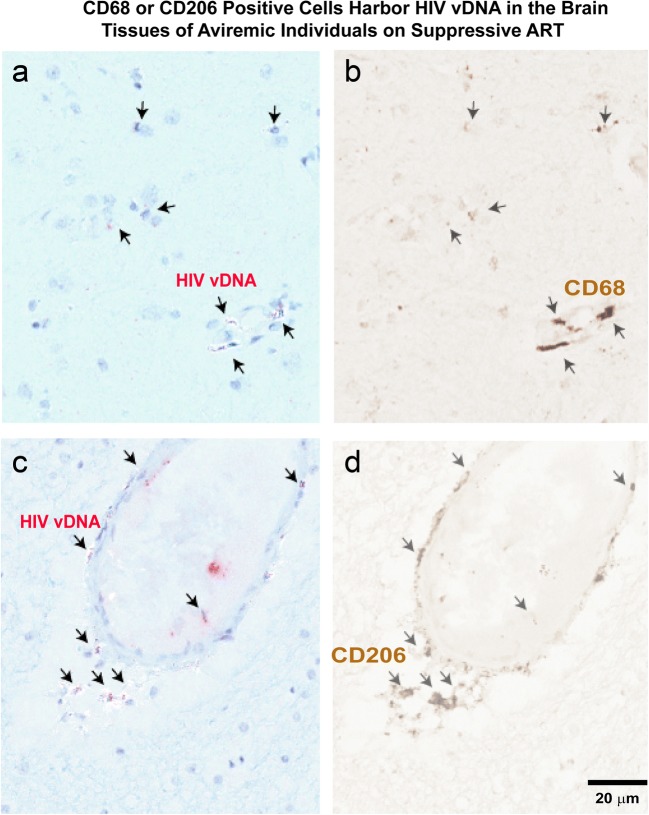
Representative images of HIV-1 DNA+ macrophages in the brain tissues from HIV infected virally suppressed aviremic individuals. HIV-1 DNA+ cell types were determined using DNAscope ISH (**a** & **c**, vDNA, red dot) in combination with IHC for a cell-type marker (**b** CD68 or **d** CD206). After being counterstained with hematoxylin, tissue sections were digitized, and a single channel image of vRNA (red) and cell-type marker (brown) were taken using Aperio’s Spectrum Plus analysis program. **a**, **b** vDNA and CD68 colocalization was indicated by black arrows, BG tissue from individual 00009, **c**, **d** vDNA and CD206 colocalization was indicated by black arrows, FWM tissue from individual 00719

**Fig. 6:**
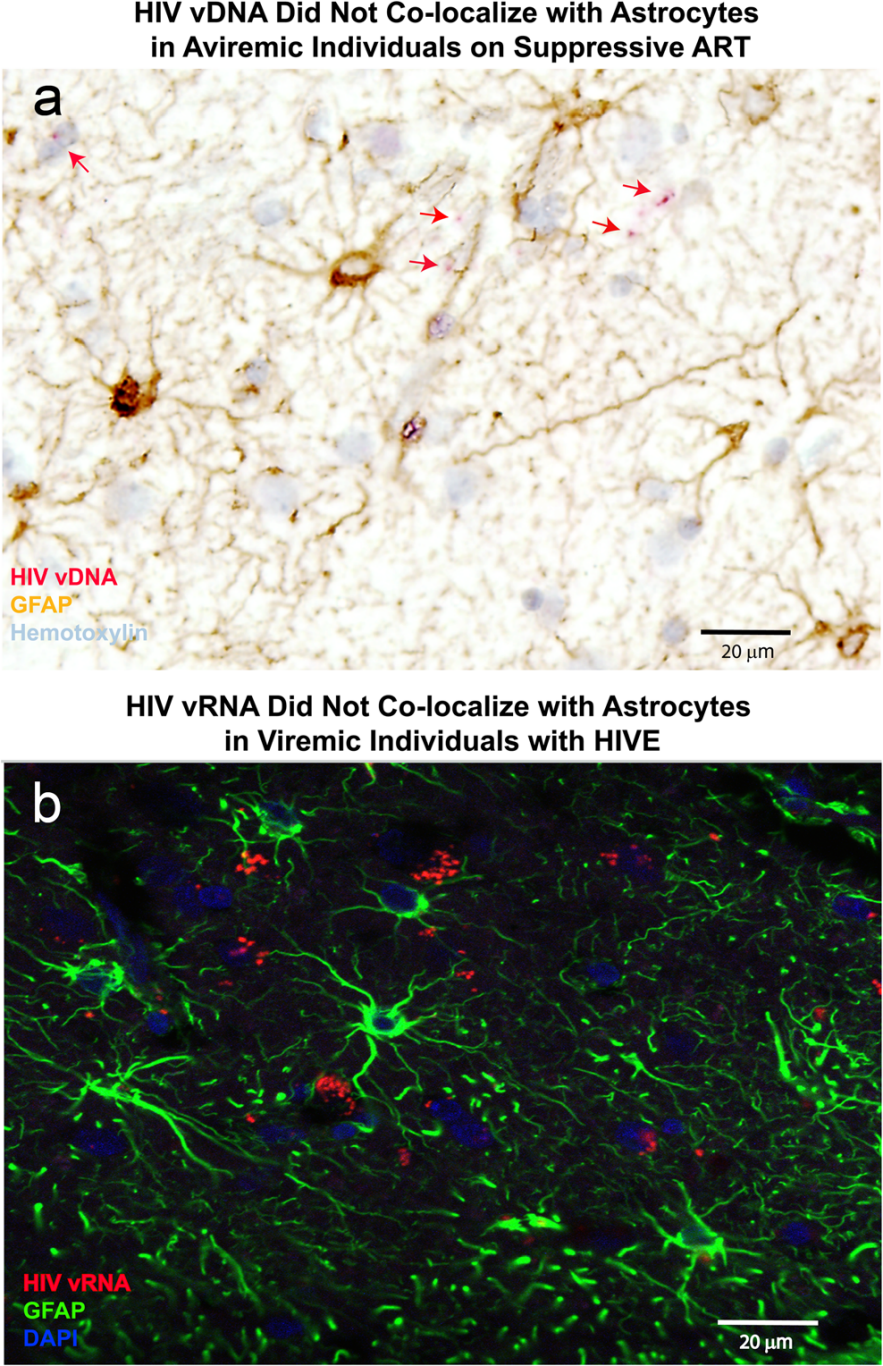
Representative images of HIV-1 vDNA and vRNA not colocalizing with astrocytes in the brain tissues of HIV-infected aviremic individuals on ART and viremic HIVE subjects respectively. **a** vDNA (red) and GFAP (brown) showed no colocalization, which was indicated by red arrows, FWM tissue from virally suppressed individual 01495. **b** vRNA (red) and GFAP (green) showed no colocalization FWM tissue from HIV-infected viremic HIVE individual 02390

Next, we sought to determine the cellular phenotype of vDNA+ cells in the brain, using DNAscope ISH in combination with IHC for CD68 (activated microglia/macrophages), CD206 (PVM) and GFAP (astrocytes). In the brain of virally suppressed subjects, HIV vDNA+ cells were found to be either CD68+ (Fig. 5a) or CD206+ (Fig. 5b), but not GFAP+ (Fig. 6a). Interestingly, many if not all vDNA+ cells are located around the vessels, suggesting, with their CD206 immunoreactivity, that they are PVM (Fabriek et al. 2005; Vogel et al. 2013; Holder et al. 2014). After we individually examined a minimum of 40 vDNA+ cells each from virally suppressed cases, we were not able to find any single vDNA+GFAP+ cells. In HIVE, cells harboring vDNA are also exclusively CD68+ microglia/macrophages (data not shown). We also examined the type of cells harboring vRNA in viremic subjects from the HIVE group, vRNA+ cells were exclusively colocalized with CD68 and CD206 (Supplementary Fig. 2a-f, black arrows). We found no evidence of the presence of vRNA in GFAP+ astrocytes in the brain with HIVE (Fig. 6b).

Recently, we demonstrated that a myeloid-specific host restriction factor SAMHD1 protein is upregulated and phosphorylated in the brain during SIV infection and correlates with levels of brain SIV DNA (Lindgren et al. 2018). In the current study, we examined SAMHD1 protein expression in the brain of HIV patients with HIVE and virally suppressed patients with or without HAND. There was a significant positive correlation between the number of SAMHD1+ nuclei and the number of HIV vDNA+ cells in the FWM (*r* = 0.5401, *p* = 0.0096) and BG (*r* = 0.6609, *p* = 0.0006) although we found no correlation in the CC (Supplementary Fig. 3).

## Discussion

Previously, using a simian immunodeficiency virus (SIV)-infected macaque model of HIV infection, Clements and colleagues showed that SIV DNA remained detectable in brain after HAART although viral replication in the brain and CSF was suppressed (Zink et al. 2010). A few brain studies examined the presence of HIV DNA by qPCR in the brains of HIV-infected, ART-treated subjects (Gelman et al. 2013; Lamers et al. 2016). However, it remains to be demonstrated which cell type(s) harbors HIV DNA and/or RNA in the brain of HIV patients receiving HAART that suppresses plasma viral load to undetectable levels (“virally suppressed patients”). The paucity of postmortem brain tissue from those who achieved viral suppression has hindered such investigations. In the present study, we are able show that cells harboring HIV-1 DNA persist in the brain despite suppressive ART and that these cells are exclusively macrophages/microglia. Moreover, we found the presence of low-level HIV-1 RNA in focal brain areas in a minority of virally suppressed patients, suggesting either spontaneous viral reactivation or ongoing low level replication despite suppressive ART. Notably, the detected vRNA morphologically resembles cell-free virions, which can infect adjacent cells to replenish reservoir. This is the first formal demonstration that HIV-1 RNA can be expressed in brains of virally suppressed patients. Therefore, the present manuscript demonstrates brain macrophages as a potential target for anti-HIV therapy. While our manuscript was under revisions, Tso et al. (2018) also reported that subtype C HIV-1 was detected by droplet digital PCR in the brains of two of four virally suppressed cases. They also demonstrated colocalization of HIV vDNA with CD68+ macrophages/microglia in one virally suppressed case by combined DNAscope ISH and IHC (Tso et al. 2018).

Interestingly, we found no evidence of the presence of vDNA or vRNA in GFAP+ astrocytes in the brain of aviremic or viremic HIV-infected patients. The role of astrocytes as an HIV reservoir remains very controversial with a few recent studies demonstrating the association of HIV-1 DNA with astrocytes (as well as neurons, in one study) using laser capture microdissection (LCM) (Trillo-Pazos et al. 2003; Churchill et al. 2009; Thompson et al. 2011). Trillo-Pazos et al. detected the presence of HIV-1 gag DNA in pooled samples of 100 astrocytes from the frontal cortical tissues of a pediatric and an adult AIDS case. Likewise, Thompson et al. detected HIV-1 gag DNA in 11 to 17% and 0 to 23% astrocytes in occipital regions of HIVE and HIV cases, respectively. However, whether astrocytes are infected remains to be proven as reactive astrocytes serving as phagocytes has been recently suggested. Churchill et al. have shown increased presence of HIV DNA in LCM-isolated astrocytes when sub-analyzed in association to proximity to PVM in AIDS cases. Furthermore, a recent study using cultured human fetal astrocytes suggests that HIV-1 DNA in astrocytes occurs by uptake of HIV-1-infected debris by cell-cell interaction with macrophages (Russell et al. 2017). Alternatively, the close spatial proximity of HIV-infected macrophages/microglia may serve as a source of contamination in examination of astrocytes. In the current study, however, we have not identified the presence of HIV-1 DNA or RNA, using single-copy sensitive RNAscope and DNAscope, respectively, in GFAP+ astrocytes in the brain of aviremic or viremic patients, which challenges the findings of HIV-1 DNA in astrocytes seen in these studies. Interestingly, in vitro studies on HIV infection of astrocytes have shown that once a block to HIV fusion and entry is artificially bypassed, there was no post-entry restriction to HIV-1 replication (Canki et al. 2001; Russell et al. 2017). The lack of intracellular restriction evolved in human primary astrocytes indicates no virus-driven selection in this cell type during primate evolution.

When comparing the three HIV groups, we noticed a trend of increased frequency of HIV-1 vDNA+ cells, specifically in the CC, from virally suppressed subjects without HAND, to subjects with HAND, and to subjects with HIVE. The above findings along with the observation of a strong positive correlation between HIV vDNA+ cells in the CC and GDS in virally suppressed subjects with HAND may suggest a potential role of HIV in the brain in the development of neurocognitive impairments despite suppressive long-term HAART. Brain imaging studies of HAART treated HIV patients have previously shown selective white matter disease (Garvey et al. 2014; Ragin et al. 2015; Randall et al. 2017; Buyukturkoglu et al. 2018) and a correlation between loss of CC integrity with neurocognitive deficits (Wu et al. 2006; Kelly et al. 2014). However, it is still unclear whether the neurocognitive impairment in the era of HAART is due to ART neurotoxicity or HIV induced neuroinflammatory responses.

In summary, this study has identified for the first time the persistence of HIV vDNA+ cells in the brain of virally suppressed patients. Using DNAscope in situ hybridization technique, we found that HIV-1 DNA is harbored exclusively in brain macrophages/microglia, especially PVM, but not astrocytes. Importantly, in a minority of virally suppressed aviremic patients, isolated and focal vRNA was detected, indicating there was a spontaneous viral reactivation and/or an ongoing low-level focal replication despite suppressive ART. Taken together, our findings suggest that brain macrophages are a important HIV reservoir.

## Electronic supplementary material

ESM 1 (XLSX 14 kb)

ESM 2 (DOCX 18947 kb)
