# Supplementary material for: Macrophages but not Astrocytes Harbor HIV DNA in the Brains of HIV-1-Infected Aviremic Individuals on Suppressive Antiretroviral Therapy
Source: J Neuroimmune Pharmacol. 2018 Sep 7;14(1):110–9. doi: 10.1007/s11481-018-9809-2 (PMC6391194; doi:10.1007/s11481-018-9809-2)
Supplement: Supplementary file 2 — (DOCX 18947 kb) [file 11481_2018_9809_MOESM2_ESM.docx]

**Ko et al., Macrophages but not astrocytes harbor HIV DNA in the brains of HIV-1-infected aviremic individuals on suppressive antiretroviral therapy**

**Online Resource**

**Supplementary Figures**


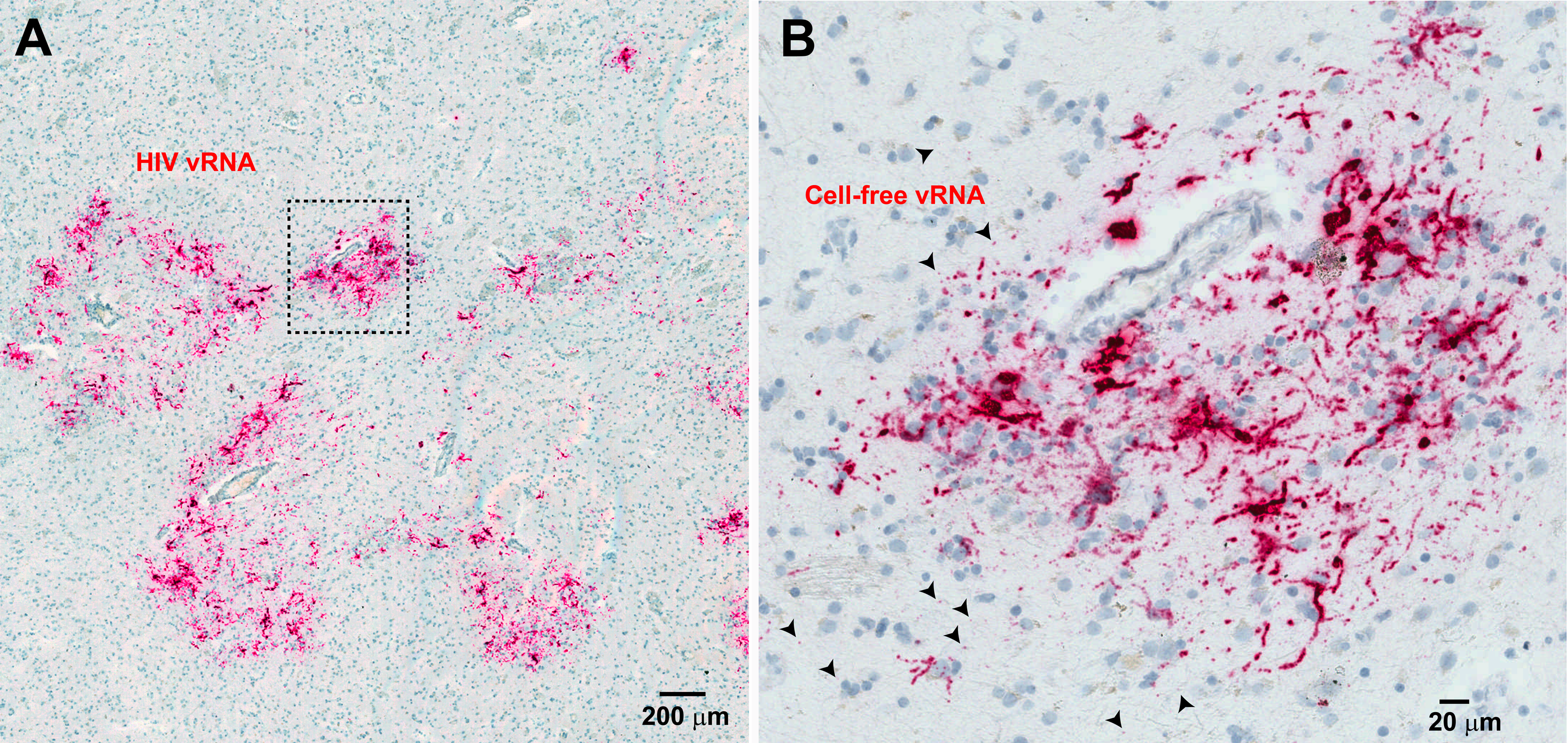


**Fig. S1** Representative images of HIV-1 RNA (vRNA) detection in the brain tissue (basal ganglia) from an HIV-infected viremic individual (MHBB519) from the HIVE group. HIV-1 vRNA (red) was detected using RNAscope in situ hybridization (ISH) with V−HIV Clade B antisense probes and RNAscope 2.5 HD red reagent kit. The cell nuclei were counterstained with hematoxylin. Scale bars are shown. (a) Low magnification, and (b) magnified image from the inset in the panel A, in which both cell-free virion derived signals (discrete red puncta, arrowheads) and cell-associated vRNA signals were visible.


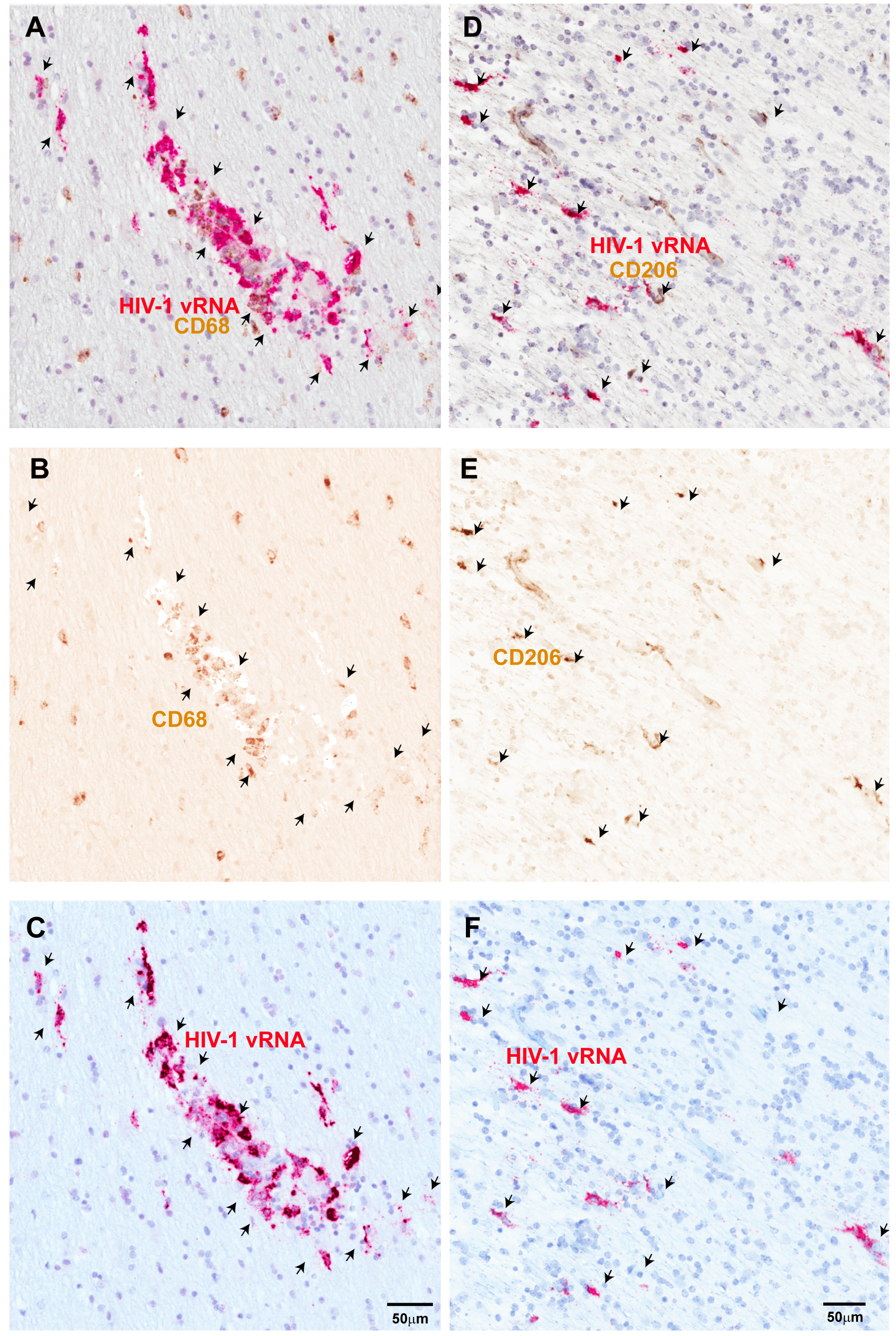


**Fig S2** Representative images of HIV-1 RNA+ cell types in the brain tissue from HIV-infected viremic individuals from HIVE group. HIV-1 RNA+ cell types were determined using RNAscope ISH (vRNA, red) in combination with immunohistochemistry (IHC) for a cell-type marker (CD68 or CD206). After being counterstained with hematoxylin, tissue sections were digitized, and a single channel image of vRNA (red, c, f), cell-type marker (brown, b, e) and in combination (a, b) were taken using Aperio’s Spectrum Plus analysis program. (a-c) vRNA and CD68 colocalization indicated by arrows, corpus callosum tissue from individual 00284, and (d-f) vRNA and CD206 colocalization indicated by black arrows, basal ganglion tissue from individual 01580.


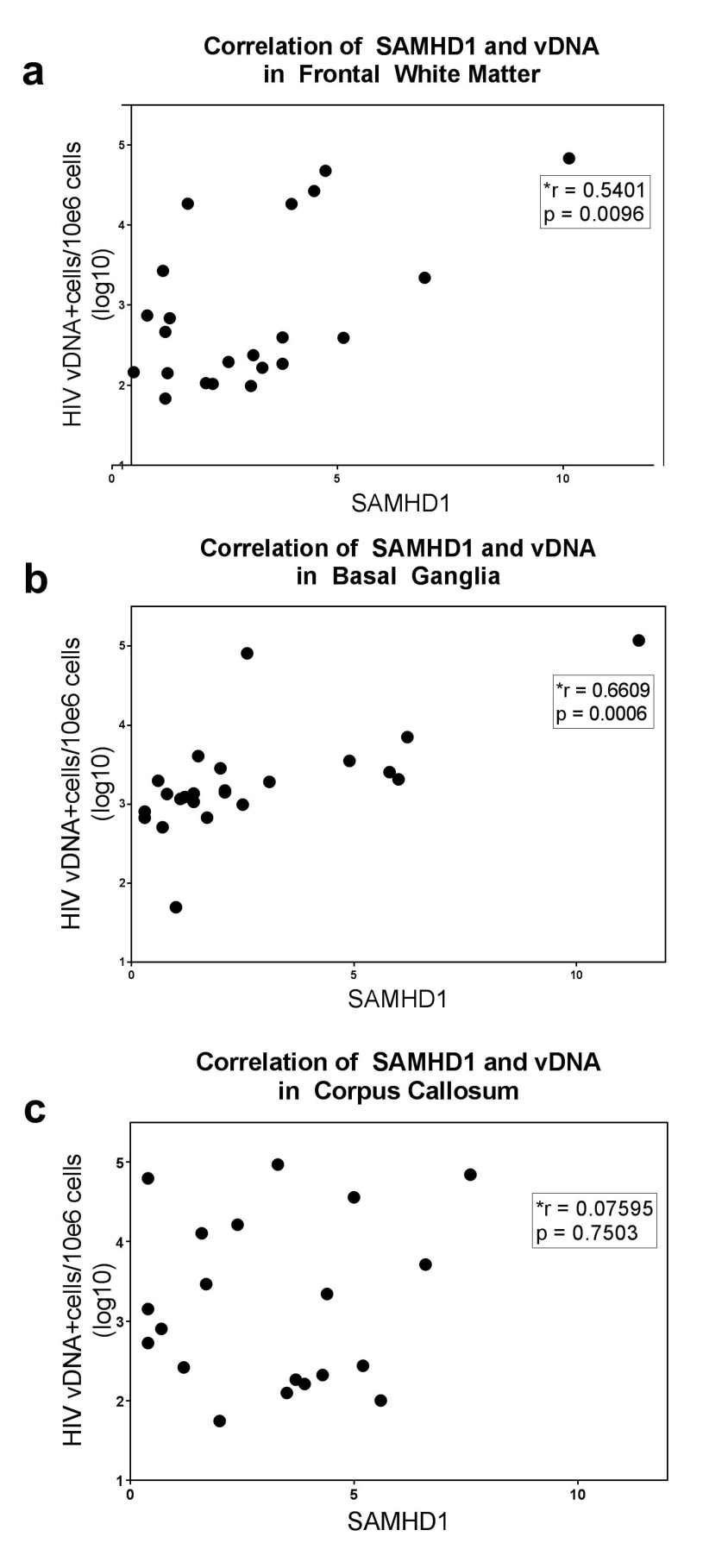


**Fig S3** HIV vDNA correlates with SAMHD1 expression in the frontal white matter (FWM) and basal ganglia (BG) of HIV-infected groups. We performed a correlation analyses between number of SAMHD1 and the number of HIV vDNA in (a) FWM, (b) BG, and (c) corpus callosum (CC). In FWM there is a positive correlation, r = 0.5401, with a p value = 0.0096. In BG there is also a positive correlation, r = 0.6609, with a p value = 0.0006. In CC, there is no significant correlation.
